# Protective Effects of the Postbiotic *Levilactobacillus brevis* BK3 against H_2_O_2_-Induced Oxidative Damage in Skin Cells

**DOI:** 10.4014/jmb.2403.03010

**Published:** 2024-05-24

**Authors:** Young-Sun Lee, Su-Jeong Lee, Won Je Jang, Eun-Woo Lee

**Affiliations:** 1 Department of Biomedicine and Pharmaceutics, Dong-Eui University, Busan 47340, Republic of Korea; 2 Research Institute for Microbiology, Dong-Eui University, Busan 47340, Republic of Korea

**Keywords:** Postbiotics, Kimchi, *Levilactobacillus brevis*, skin damage

## Abstract

Postbiotics have various functional effects, such as antioxidant, anti-inflammatory, and anti-obesity. *Levilactobacillus brevis* BK3, the subject of this study, was derived from lactic acid bacteria isolated from Kimchi, a traditional Korean fermented food. The antioxidant activity of BK3 was confirmed through the measurements of 2,2-diphenyl-1-picryl-hydrazyl (DPPH), 2,2'-azino-bis (3-ethylbenzothiazoline-6-sulfonic acid) (ABTS), and total antioxidant capacity (TAC). The wrinkle improvement effect was validated by assessing elastase inhibitory activity and collagenase inhibitory activity. The intracellular activity was confirmed using human keratinocytes (HaCaT) and human fibroblasts (HFF-1). BK3 protects skin cells from oxidative stress induced by H_2_O_2_ and reduces intracellular reactive oxygen species (ROS) production. In addition, the expressions of the antioxidant genes superoxide dismutase (SOD), catalase (CAT), and glutathione peroxidase (GPx) were upregulated. Meanwhile, matrix metalloproteinase-1 (MMP-1) and collagen type I alpha 1 (COL1A1), involved in collagen degradation and synthesis, were significantly regulated. These results suggest the possibility of utilizing BK3 as a functional ingredient with antioxidant and wrinkle-improving effects.

## Introduction

Postbiotics are preparation of inanimate microorganisms and/or their components, which provide health benefits to the host [1]. Postbiotics include bacteria lysate, cell free supernatants, cell wall fragments, short-chain fatty acids (SCFA), exopolysaccharide (EPS), enzymes, etc., and can be produced by various methods such as high temperature, high pressure, and sonication [2, 3]. Postbiotics are relatively safer than probiotics because they are dead cells or metabolites and have the advantage of being easily transported and stored during production, maintaining stable quality [4]. Postbiotics are also used in skin research and are reported to have various effects, such as alleviating inflammation, whitening, and preventing moisture loss [5-10].

The skin is the organ that occupies the largest area in the body and is composed of the epidermis, dermis, and subcutaneous layers [11]. Skin function deteriorates with age, and skin aging is caused by endogenous factors, such as genetics, hormones, and metabolic processes, and exogenous factors, such as UV, chemicals, and pollution [12, 13]. When skin aging occurs, the thickness of the epidermis and dermis decreases, and the barrier that protects the body from the outside deteriorates. As fibroblasts age, symptoms such as skin dryness, loss of elasticity, and wrinkles occur [14, 15]. When the skin is exposed to external stimuli, reactive oxygen species (ROS) are generated, and ROS, such as superoxide anion (·O_2_^-^), hydroxyl radical (·OH), and hydrogen peroxide (H_2_O_2_) induce cell death [16]. An appropriate amount of ROS helps maintain intracellular homeostasis and signal transmission, but excessive accumulation of ROS within cells induces cell damage, resulting in skin aging and inflammation [17]. ROS generated in the body are removed by antioxidants, and the nuclear factor erythroid-2-related factor 2 (Nrf2) pathway, one of the antioxidant mechanisms in the body. The Nrf2 pathway activities antioxidant response elements (ARE) when oxidative stress occurs and regulates the activity of antioxidant enzymes, such as catalase (CAT), superoxide dismutase (SOD), glutathione S-transferase (GST), and heme oxygenase-1 (HO-1) [18-20]. This study evaluated the antioxidant and wrinkle-improving activities of the postbiotic *Levilactobacillus brevis* BK3, isolated from Kimchi.

## Materials and Methods

### Bacteria Isolation and Identification

Bacteria were isolated from bean leaf Kimchi. Kimchi and phosphate-buffered saline (PBS) were mixed at 1:9 (w/v), homogenized using a stomacher, and then serially diluted. Each suspension was cultured in De Man, Rogosa, and Sharpe (MRS; MBcell, Republic of Korea) medium at 37°C for 24 h, and single colonies were separated. Identification of the isolated strains was analyzed through 16S rRNA sequencing using 27F (5'-AGA GTT TGA TCM TGG CTC AG-3') and 1492R (5'-GGT TAC CTT GTT ACG T-3') primer sets.

### Preparation of Postbiotics

Bacteria at a concentration of 10^9^ CFU/ml were obtained by culturing in 5 ml of MRS broth at 37°C for 24 h. The cultured bacteria were centrifuged (4,000 rpm, 4°C, 10 min) to remove the supernatant and washed twice with the same volume of PBS. The bacteria were then treated with 1 mg/ml lysozyme (Sigma, USA) and reacted at 37°C for 30 min. Afterwards, cells were disrupted using a sonicator (Ultrasonics Sonifier 250; Branson Ultrasonics, USA) and centrifuged (4,000 rpm, 4°C, 10 min) to remove bacterial debris and obtain a clear supernatant. The harvested supernatant was spread on MRS agar and it was confirmed that bacteria did not grow. The completed postbiotics solution was aliquot and stored at -20°C until used in the experiments.

### Cell Culture

Human epidermal keratinocyte HaCaT cells (Addexbio, USA) were cultured in Dulbecco’s modified Eagle’s medium (DMEM; WELGENE, Republic of Korea) supplemented with 10% fetal bovine serum (FBS; HyClone, USA) and 1% penicillin and streptomycin (P/S; Gibco, USA). Human foreskin fibroblast HFF-1 cells (ATCC, USA) were incubated in DMEM supplemented with 15% FBS and 1% P/S. All cells were cultured at 37°C with 5% CO_2_.

### DPPH Radical Scavenging Activity

The radical scavenging activity of 2,2-diphenyl-1-picrylhydrazyl (DPPH) was determined using the method described by Das *et al*. [21], with a few modifications. DPPH was dissolved in ethanol to make a 0.2 mM DPPH solution, then mixed with postbiotics 1:1 (v/v) and reacted at 37°C for 30 min, protected from light. The absorbance was determined at 517 nm using a microplate spectrophotometer (BioTek Synergy HTX; BioTek, USA). For a positive control 0.1 mM L-ascorbic acid was used, and the same amount of distilled water was used as a control instead of postbiotics. DPPH radical scavenging was calculated using the following equation:

$$\text{DPPH radical scavenging activity (\%) =}\left(1-\frac{\text{Absorbance of sample}}{\text{Absorbance of control}}\right)\times 100$$

### ABTS Radical Scavenging Activity

The radical scavenging activity of 2, 2’-azino-bis (3-ethylbenzothiazoline-6-sulfonic acid) (ABTS) was determined using the method described by Afify *et al*. [22], with a few modifications. The ABTS solution was prepared by mixing 2.4 mM potassium persulfate and 7.4 mM ABTS at a ratio of 1:1 (v/v) and reacting for 16 h at room temperature, protected from light. The ABTS solution was used by adjusting the absorbance value to 0.7 ± 0.05 at 734 nm just before use. The ABTS solution (180 ul) and postbiotics (20 ul) were mixed and reacted at room temperature for 6 min, then the absorbance was determined at 734 nm. For a positive control, 5 mM trolox was used, and the same amount of distilled water was used as a control instead of the postbiotics. ABTS radical scavenging activity was calculated using the following equation:

$$\text{ABTS radical scavenging activity (\%) =}\left(1-\frac{\text{Absorbance of sample}}{\text{Absorbance of control}}\right)\times 100$$

### Total Antioxidant Capacity (TAC)

The TAC (Total Antioxidant Capacity) of the postbiotics was determined using an EZ-TAC assay kit (DoGenBio, Republic of Korea). According to the manufacturer’s protocol, 100 ul of postbiotics was mixed with 100 ul of the copper reagent and 100 ul of the reaction buffer, reacted at room temperature for 30 min, and the absorbance was determined at 450 nm. The antioxidant capacity of the postbiotics was expressed as the trolox equivalent (TE) using the standard curve of trolox.

### Elastase Inhibitory Activity

Elastase inhibitory activity was determined according to the method by Shirzad *et al*. [23], with some modifications. N-succinyl-ala-ala-ala-p-nitroanilide (30 ul), postbiotics (50 ul), and elastase (10 ul) (0.6 U/ml) were mixed in 100 ul of 0.2 M Tris-HCl buffer (pH 8.0) and reacted at 37°C for 20 min. The absorbance was then measured at 410 nm. As a positive control, 0.1 mM oleanolic acid was used, and the same amount of 0.2 M Tris-HCl buffer was used as a control instead of the postbiotics. The elastase inhibitory activity was calculated using the following equation:

$$\text{Elastase inhibitory activity (\%) =}\left(1-\frac{\text{Absorbance of sample}}{\text{Absorbance of control}}\right)\times 100$$

### Collagenase Inhibitory Activity

Collagenase inhibitory activity was analyzed using a Collagenase Activity assay kit (Abcam, UK). The experiment was conducted in accordance with the manufacturer’s protocol. After mixing 10 ul of collagenase and 88 ul of assay buffer with 2 ul of the sample, 100 ul of the reaction buffer was added. The absorbance of each well was immediately measured at 345 nm for 15 min in kinetic mode. For a positive control, 1 M of 1,10-phenanthroline was used.

### Cell Viability Assay

Cell viability was measured using a Viability assay kit (WST-8, Cellrix, Republic of Korea). Cells were cultured in a 96-well cell culture plate and incubated for 24 h at 37°C with 5% CO_2_. The cells were treated with 0.1% to 3% (v/v) BK3 or 700 uM to 900 uM H_2_O_2_ in DMEM without FBS and antibiotics. Subsequently, the cells were incubated with 10 ul of the WST-8 solution and 100 ul of serum-free DMEM at 37°C for 2 h, the absorbance was measured at 450 nm. Cell viability was calculated using the following equation:

$$\text{Cell viability (\%) =}\left(\frac{\text{Absorbance of sample-Absorbance of blank}}{\text{Absorbance of control-Absorbance of blank}}\right)\times 100$$

### Determination of Intracellular ROS Production

Levels of intracellular ROS were measured using a DCFDA/H2DCFDA-cellular ROS assay kit (Abcam). Cells were seeded in a 96-well cell culture plate and incubated for 24 h. The cells were pretreated with BK3 diluted in DMEM without FBS and antibiotics. After treatment, 20 uM of the DCFDA solution was added, and the reaction was performed at 37°C for 45 min. Afterward, H_2_O_2_ was added to the wells. The fluorescence intensity was measured at excitation and emission wavelengths of 485 nm and 528 nm, respectively.

### mRNA Expression Analysis Using RT-qPCR

To measure gene expression, cells were treated with various concentrations of BK3 and cultured. The cells were then treated with H_2_O_2_ to induce oxidative stress. Total RNA was extracted using a Hybrid-R Kit (GeneAll, Republic of Korea) according to the manufacturer's instructions. The concentration and purity of the extracted RNA were determined using NanoVue (GE Healthcare, USA), and cDNA was synthesized using a PrimeScript 1st strand cDNA Synthesis Kit (Takara, Japan). Real-time quantitative polymerase chain reaction (RT-qPCR) was performed using TB Green Premix Ex Taq (Takara) reagent and CFX96 (Bio-Rad, USA) at Core-Facility Center for Tissue Regeneration (Dong-eui University, Republic of Korea). The expression levels of SOD, CAT, GPx, MMP-1, and COL1A1 were calculated using the 2^-ΔΔCt^ method, and the relative quantification was performed based on glyceraldehyde 3-phosphate dehydrogenase (GAPDH). The primer sequences used in the RT-qPCR are listed in Table 1.

### Statistical Analysis

All experiments were performed in triplicate, and the experimental results are expressed as the mean ± standard deviation. IBM's Statistical Package for the Social Sciences software (SPSS Inc., version 26.0, USA) was used to perform independent samples student’s *t*-test and one-way ANOVA. The significance of each treatment group was analyzed using Duncan’s multiple range test, and statistical significance was indicated by a *p*-value less than 0.05. The graphs were generated using GraphPad Prism 10 software (GraphPad Software Inc.,USA).

## Results

### Bacteria Identification

**Fig. 1:**
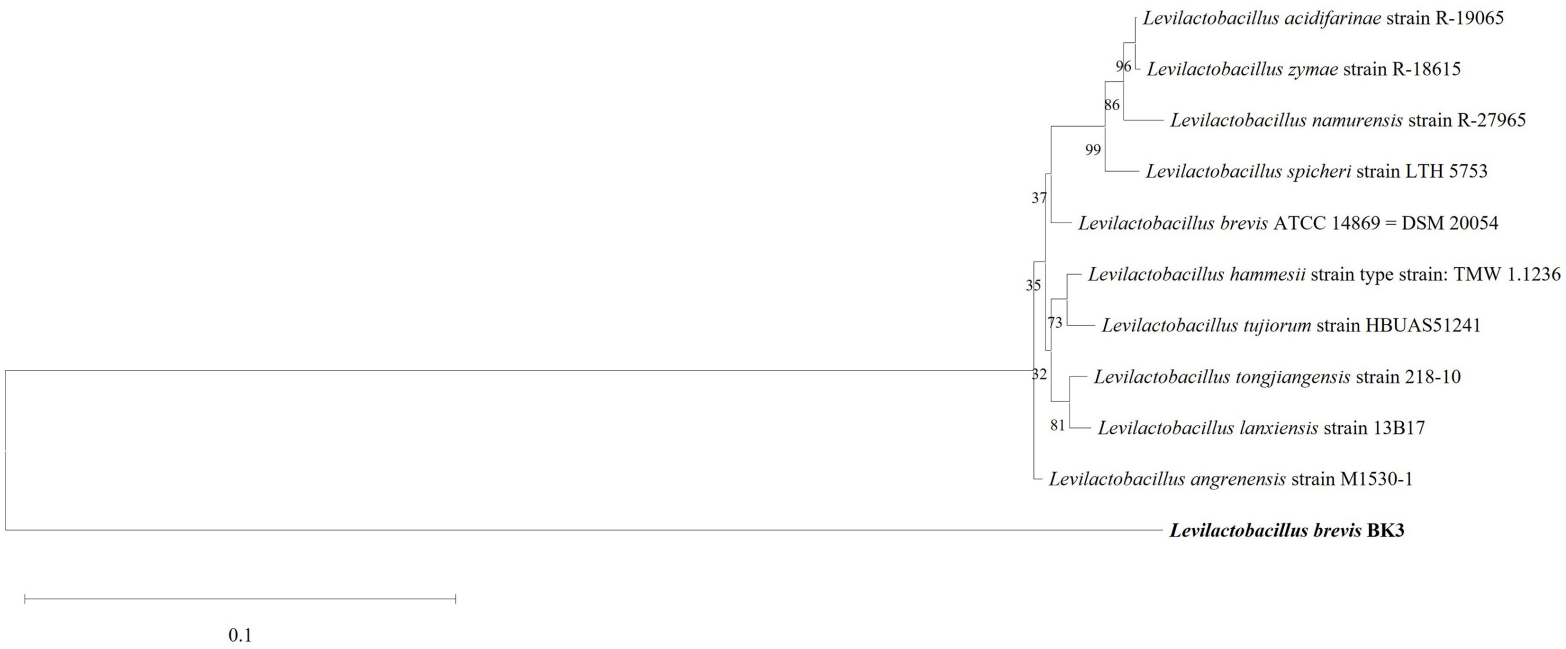
Phylogenetic tree of *L. brevis* BK3 isolated from bean leaf Kimchi, analyzed based on 16S rRNA gene sequences. The phylogenetic tree was constructed using the neighbor joining method using MEGA 11 with 1,000 bootstrap repetitions, after which the 16S rRNA sequences were aligned using the Clustal W program. BK3, *L. brevis* BK3.

Isolated bacteria were identified using 16S rRNA sequencing analysis. The 16S rRNA sequences of the isolated bacteria shared 99.87%, 99.62%, and 99.36% homology with *L. brevis* ATCC 14869 = DSM 20054 (NR_116238.1), *L. angrenensis* strain M1530-1 (NR_180286.1), and *L. spicheri* strain LTH 5753 (NR_025579.1), respectively (Fig. 1). The isolated strain was named *L. brevis* BK3.

### Antioxidant Activities of Postbiotics

The antioxidant activity of BK3 was determined by DPPH and ABTS radical scavenging activities and a TAC assay (Table 2). The scavenging activity of L-ascorbic acid, used as a positive control for DPPH radical scavenging activity, was 90.70 ± 0.35%, and the scavenging activity of trolox, used as a positive control of ABTS radical scavenging activity, was 24.12 ± 1.16%. The scavenging activity of *Lacticaseibacillus rhamnosus* GG was found to be 24.12 ± 1.16% and 30.96 ± 1.20% for DPPH and ABTS radicals, respectively. The DPPH and ABTS radical scavenging activities of BK3 were 30.97 ± 5.56% and 46.65 ± 0.76%, respectively, and had superior activity compared with *L. rhamnosus* GG. The TAC was expressed as a TE value, and the TE value of BK3 was 13.24 ± 1.93 uM/10 ul, which was higher than that of *L. rhamnosus* GG, which was 5.98 ± 1.93 uM/10 ul.

### Anti-Wrinkle Activities of Postbiotics

The anti-wrinkle activity of BK3 was determined by elastase and collagenase inhibitory activities (Table 3). Oleanolic acid, used as a positive control for elastase inhibition, had an inhibitory activity of 68.83 ± 2.67%, and *L. rhamnosus* GG had an inhibitory activity of 41.30 ± 4.15%. The inhibition effect of BK3 was relatively higher at 50.24 ± 3.12%. As a positive control for collagenase inhibitory activity, 1,10-phenanthroline was used and had an inhibitory activity of 100.56 ± 4.36%. The inhibitory activity of BK3 was found to be 14.37 ± 7.92% and was similar to *L. rhamnosus* GG, which had an inhibitory ability of 18.42 ± 3.10%.

### Effects of Postbiotics against Oxidative Stress in HaCaT Keratinocytes

**Fig. 2:**
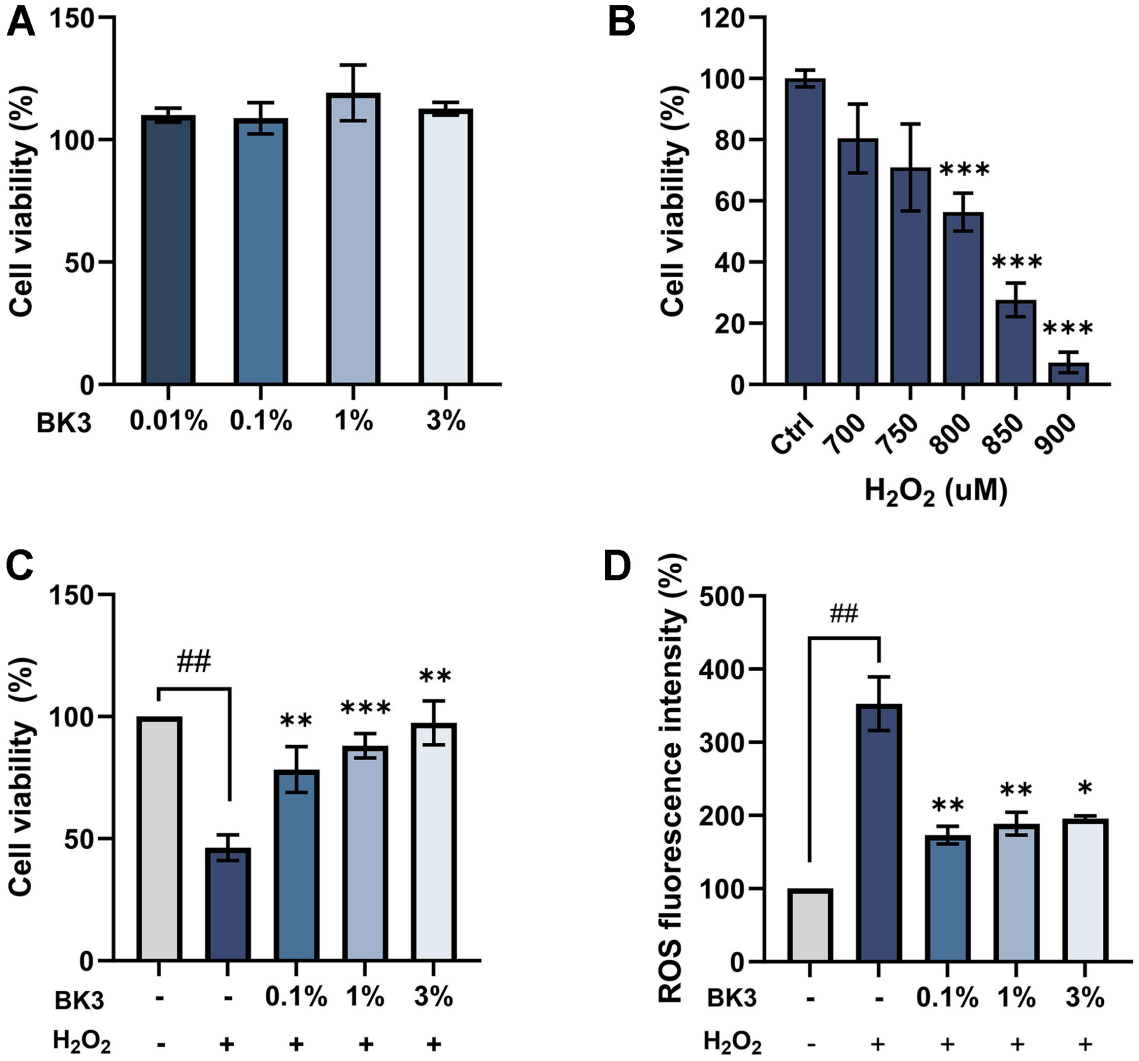
Effects of *L. brevis* BK3 in HaCaT cells. (**A**) Cytotoxicity by concentration of *L. brevis* BK3. (**B**) Changes in cell viability depending on H_2_O_2_ concentration. (**C**) Cell protective activity against oxidative stress. (**D**) Intracellular ROS reduction due to BK3 treatment. All values are the mean ± SD. Statistically significant differences are indicated as *p < .05, **p < .01, and ***p < .001. BK3, *L. brevis* BK3.

To confirm cytotoxicity, HaCaT cells were treated with BK3 at different concentrations (0.01% to 3%). Cell viability was checked, and no cytotoxicity was observed at all concentrations (Fig. 2A). To induce oxidative stress in HaCaT cells, H_2_O_2_ was used at different concentrations. When cells were treated with 800 uM H_2_O_2_, cell viability was decreased by about 50% (Fig. 2B) [28]. After pretreating cells with BK3, the cytoprotective effect against oxidative stress was confirmed by treating the cells with H_2_O_2_. When pretreated with BK3, the cell viability was increased to 97.48 ± 8.99%, which represents a significant difference compared with the group treated with H_2_O_2_ alone (Fig. 2C). Intracellular ROS was increased more than 3-fold when cells were treated with H_2_O_2_ alone. When cells were pretreated with BK3, intercellular ROS was decreased to 173.07 ± 12.02% in the 0.1% treatment group (Fig. 2D).

### Effects of Postbiotics against Oxidative Stress in HFF-1 Fibroblasts

**Fig. 3:**
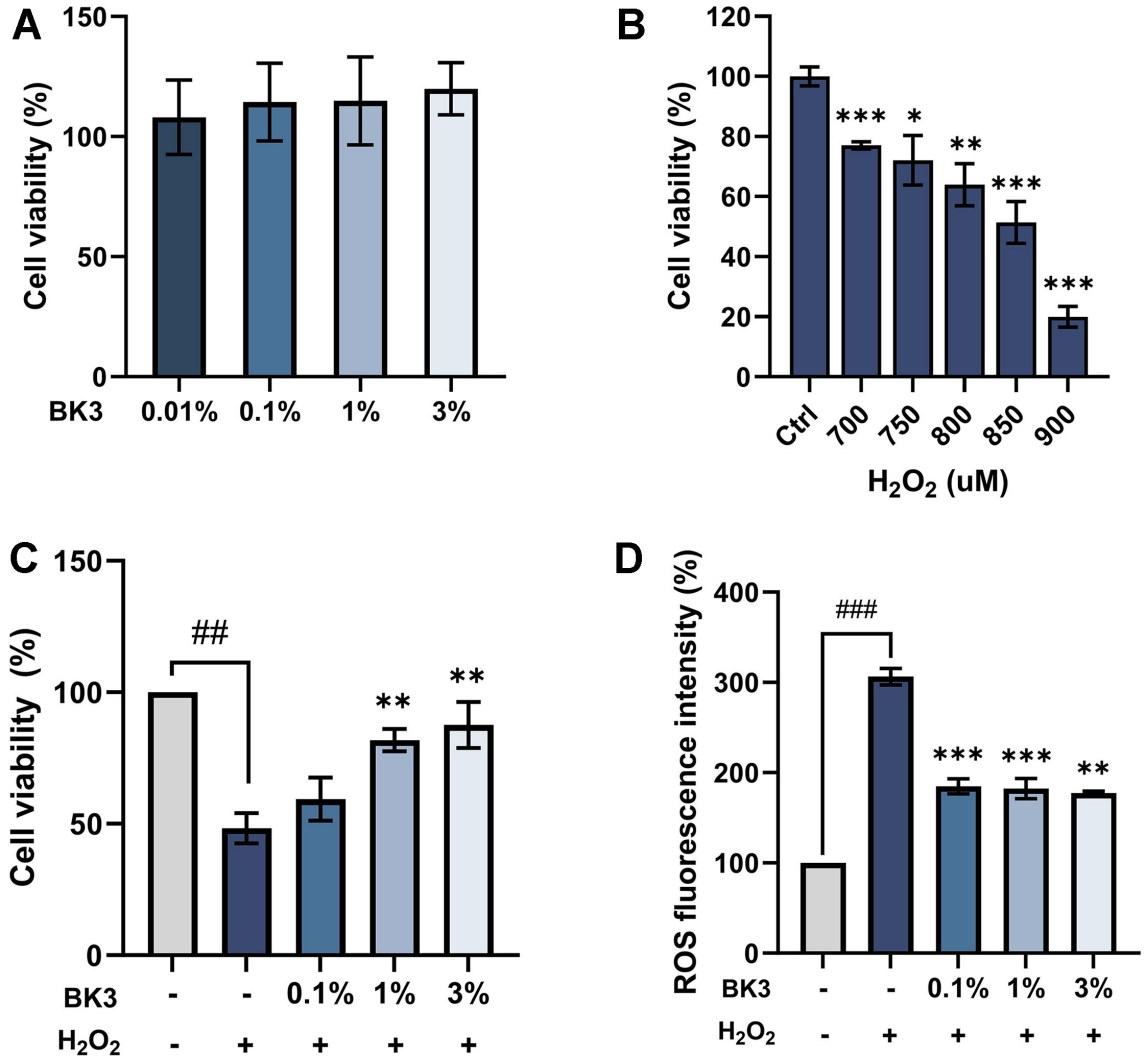
Effects of *L. brevis* BK3 in HFF-1 cells. (**A**) Cytotoxicity by concentration of *L. brevis* BK3. (**B**) Changes in cell viability depending on H_2_O_2_ concentration. (**C**) Cell protective activity against oxidative stress. (**D**) Intracellular ROS reduction due to BK3 treatment. All values are the mean ± SD. Statistically significant differences are indicated as *p < .05, **p < .01, and ***p < .001. BK3, *L. brevis* BK3.

HFF-1 cells were treated with BK3 at different concentrations (0.01% to 3%) to confirm cytotoxicity. As a result, no cytotoxicity was observed at any concentration of BK3 (Fig. 3A). Cells were treated with H_2_O_2_ at various concentrations to induce oxidative stress. The results showed that the treatment with 850 uM of H_2_O_2_ reduced cell viability by approximately 50% (Fig. 3B) [28]. The cytoprotective effect against oxidative stress was confirmed by pretreating cells with BK3 and then treating them with H_2_O_2_. When cells were treated with H_2_O_2_ alone, the cell viability was decreased by about 50%. In comparison, when cells were pretreated with BK3 at a concentration of 3%, the cell viability was increased to 87.55 ± 8.79% (Fig. 3C). Intracellular ROS was confirmed through a fluorescence intensity measurement, and the fluorescence intensity when cells were treated with H_2_O_2_ was 306.35± 9.11%. When cells were pretreated with BK3, the fluorescence intensity was relatively lower at 177.44 ± 1.92%(Fig. 3D).

### Effects of Postbiotics on Regulation of Gene Expression in Skin Cells

**Fig. 4:**
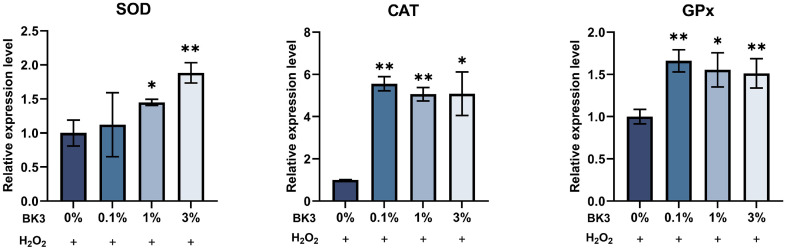
Antioxidant-related gene expressions in HaCaT cells. All values are the mean ± SD. Statistically significant differences are indicated as *p < .05, **p < .01, ***p < .001. BK3, *L. brevis* BK3; SOD, superoxide dismutase; CAT, catalase; GPx, glutathione peroxidase.

**Fig. 5:**
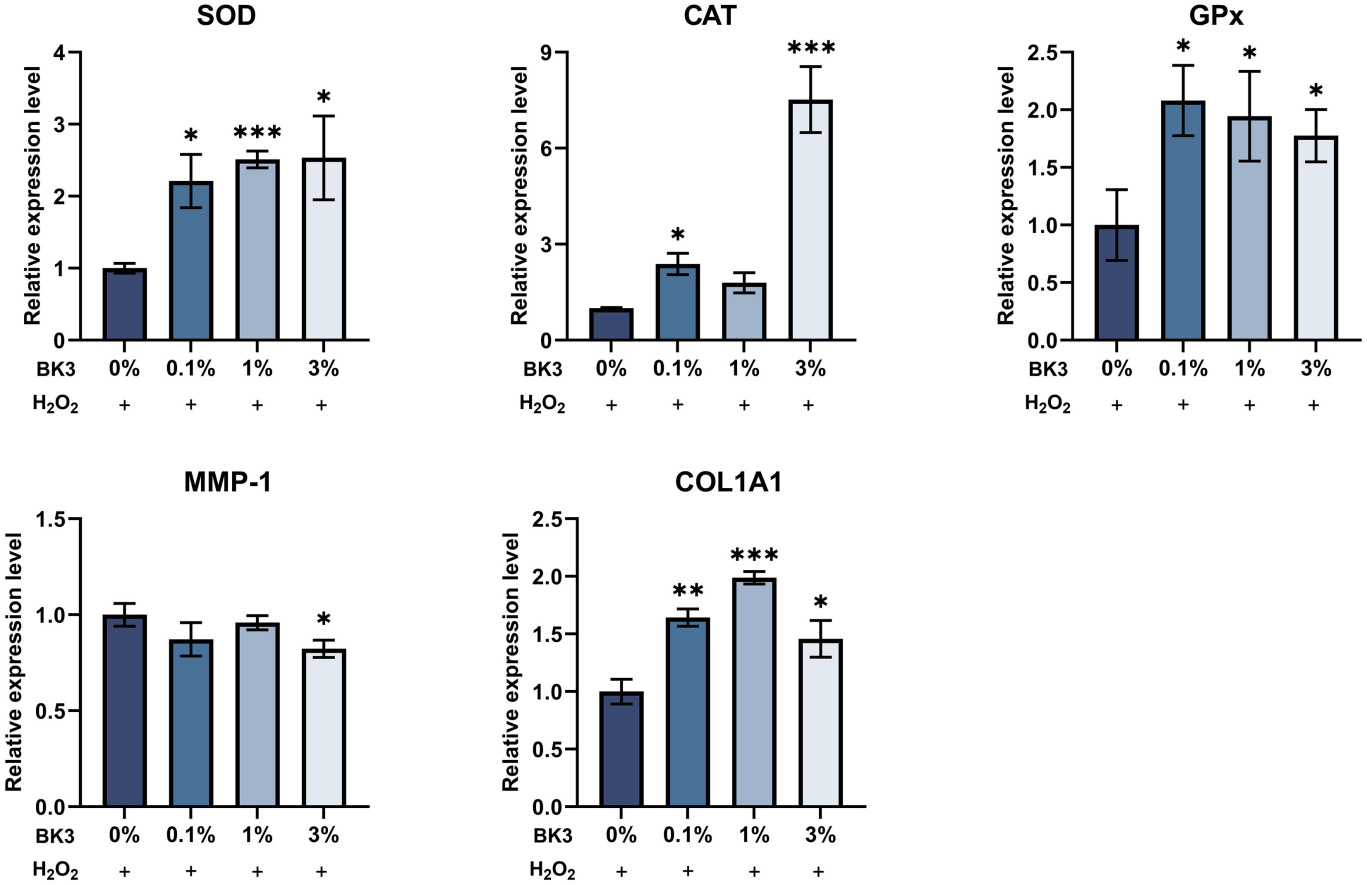
Antioxidant and wrinkle-related gene expressions in HFF-1 cells. All values are the mean ± SD. Statistically significant differences are indicated as *p < .05, **p < .01, ***p < .001. BK3, *L. brevis* BK3; SOD, superoxide dismutase; CAT, catalase; GPx, glutathione peroxidase; MMP-1, matrix metalloproteinase-1; COL1A1, collagen type I alpha 1.

After treating skin cells with BK3 for a certain period, oxidative stress was induced, and changes in mRNA expressions were analyzed. As a result, BK3 significantly regulated the expression of antioxidant and wrinkle-related genes within skin cells. When HaCaT cells were treated with BK3, the expression of SOD was significantly increased at concentrations of 1% or more. The expressions of CAT and GPx also had significant increases compared with the group treated with H_2_O_2_ alone at all concentrations (Fig. 4). When HFF-1 cells were treated with BK3, the expressions of SOD and GPx were significantly increased at all concentrations. The expression of CAT had a significant difference at concentrations of 0.1% and 3% (Fig. 5). In HFF-1 cells, the expression of MMP-1 was significantly decreased upon treatment with 3% BK3. The expression of COL1A1 had significant differences at all concentrations compared with the group treated with H_2_O_2_ alone.

## Discussion

BK3 corresponds to a bacterial lysate among the various types of postbiotics. Bacterial lysates have been reported to have various effects related to skin health, such as skin moisturizing effect, improvement of skin barrier function, and whitening [29-31]. This study confirmed the antioxidant and anti-wrinkle effects of the postbiotic BK3.

Oxidative stress is caused by the production of ROS and an imbalance in antioxidant capacity, so increasing antioxidant capacity could help reduce oxidative stress [32]. In this study, experiments were conducted to evaluate the antioxidant activity of BK3, and the well-known antioxidants L-ascorbic acid and trolox were used as positive control [33, 34]. The antioxidant activity of BK3 did not reach that L-ascorbic acid and trolox. However, in a previously reported study, the DPPH radical scavenging activity of the postbiotic *Lacticaseibacillus paracasei* DCF0429 was found to be 26.27 ± 1.85% and the ABTS radical scavenging activity of postbiotic prepared form *L. plantarum* was found to be 18.11 to 38.64% [35, 36]. In comparison, the DPPH and ABTS radical scavenging activities of BK3 were 30.97 ± 5.56% and 46.65 ± 0.76%, respectively, showing relatively high antioxidant capacity and expected to be helpful in suppressing oxidative stress.

Among the various ROS, H_2_O_2_ is generated from almost all oxidative stress and oxygen radicals and can diffuse freely in and out of cells and tissues [37]. Additionally, H_2_O_2_ changes ion homeostasis by regulating signal transduction pathways and, as a result, activates transcription factors through the expression of several genes, causing cell proliferation and differentiation [38]. H_2_O_2_, which has these functions, ultimately induces cell death by apoptosis or necrosis in various cells [39, 40]. Considering that a relatively high cell survival rate was observed when oxidative stress was induced with H_2_O_2_ after the pretreatment with BK3, it is believed that postbiotic treatments might protect cells from oxidative stress, suppressing cell death.

Decreases in collagen, elastin, and hyaluronic acid produced from fibroblasts affect the development of skin wrinkles [40]. ROS activates proteolysis of the dermal extracellular matrix (ECM) and increases the breakdown of collagen and elastin, thereby reducing skin elasticity and causing wrinkles [41]. Also, when ROS accumulates excessively in the skin due to oxidative stress, aging-related symptoms, such as deterioration of skin functions and roughening of the skin surface, can cause inflammation [42]. Compared to the previous study showing that postbiotics prepared to *L. brevis* Y3-4 had an elastase inhibitory activity of about 20%, BK3 in this study showed a relatively high activity (50.24 ± 3.12%), then BK3 is expected to be effective in improving wrinkles [23]. In addition, treating skin cells with BK3 reduced the amount of ROS generated due to oxidative stress and might effectively prevent skin elasticity loss and wrinkles by inhibiting ECM decomposition.

When oxidative stress occurs, the body's antioxidant system removes ROS and protects the cells [43]. In the skin, various antioxidants prevent skin damage caused by ROS and prevent aging [44]. The antioxidant enzymes SOD, CAT, and GPx are the primary antioxidants that are produced in the body and have relatively strong antioxidant effects [45]. SOD decomposes ·O_2_^-^ into H_2_O_2_ and O_2_ to prevent lipid peroxidation and DNA damage [46]. CAT protects cells from oxidative stress by breaking down H_2_O_2_ into H_2_O and O_2_ and prevents the accumulation of ROS [47]. GPx decomposes H_2_O_2_ and maintains cell function by regulating intracellular redox balance [48]. However, the functions of the antioxidant enzymes generated in the body gradually decrease with age [45]. Therefore, it is important to increase the activity of antioxidant enzymes. It has been reported that postbiotics prepared from *L. paracasei* increase the expression of Cu/Zn-SOD, GPx, and GST and are effective in improving damage caused by oxidative stress [49]. BK3 significantly regulated the expression of antioxidant genes in keratinocytes and fibroblasts, so it could increase intracellular antioxidant activity and protect cells from oxidative stress. ROS generated due to oxidative stress increases the expression of matrix metalloproteinase (MMP), which decomposes the ECM [49]. In particular, MMP-1 is known to significantly impact skin aging because it decomposes type I collagen and type III collagen, which account for the largest proportion of skin collagen [50, 51]. BK3 is expected to be effective in suppressing intracellular collagen degradation, as it significantly reduced the expression of MMP-1 at a concentration of 3%. In addition, BK3 upregulated the expression of COL1A1, which synthesizes collagen. These results indicate that BK3 could help improve wrinkles and maintain skin structure.

The results of this study confirmed that BK3 protects skin cells from oxidative stress, inhibits cell death, and regulates the expression of antioxidant and wrinkle related genes. These results suggest the possibility that BK3 can be used as a functional raw material. Postbiotics are suitable for use as skin care ingredients because their chemical composition can be defined, not transmitting antibiotic resistance, so they can be used even in patients with weakened immunity and having a long shelf life [52]. In fact, postbiotics are already being used as ingredients in skin care products. Topical skincare lotions containing postbiotics have a positive effect in the treatment of acne by improving hydration and regulating the skin’s pH [53]. Cream containing *Bacillus coagulans* postbiotics is effective in relieving acne through antibacterial action and reduction of sebum secretion [54]. Therefore, it is expected that postbiotics BK3 can also be used as an ingredient in products that help skin health.

## Figures and Tables

**Table 1:** Primer sequences used in the study.

Gene		Sequences (5' to 3')	Ref.
GAPDH	F	CATCTTCTTTTGCGTCGCCA	[24]
	R	TTAAAAGCAGCCCTGGTGACC	
SOD1	F	AGGCATGTTGGAGACTTGGG	[25]
	R	TTCATGGACCACCAGTGTGC	
CAT	F	TCACACCTTTGTGCAGTCCG	This study
	R	GGGTTACACGGATGAACGCT	
GPx	F	CCAGTTTGGGCATCAGGAGAA	[26]
	R	CGAAGAGCATGAAGTTGGGCT	
MMP-1	F	AGTGGCCCAGTGGTTGAAAA	This study
	R	CCACATCAGGCACTCCACAT	
COL1A1	F	GAGGGCCAAGACGAAGACATC	[27]
	R	CAGATCACGTCATCGCACAAC	

**Table 2:** Antioxidant activity of *L. brevis* BK3.

Samples	Antioxidant activity		
	DPPH radical scavenging (%)	ABTS radical scavenging (%)	TE (uM/10 ul)
Positive control	90.70 ± 0.35^a^	89.06 ± 0.89^b^	-
*L. rhamnosus* GG	24.12 ± 1.16	30.96 ± 1.20	5.98 ± 0.86
*L. brevis* BK3	30.97 ± 5.56	46.65 ± 0.76	13.24 ± 1.93

^a^DPPH radical scavenging of L-ascorbic acid.

^b^ABTS radical scavenging of trolox.

**Table 3:** Anti-wrinkle activity of *L. brevis* BK3.

Samples	Anti-wrinkle activity	
	Elastase inhibitory (%)	Collagenase inhibitory (%)
Positive control	68.83 ± 2.67^a^	100.56 ± 4.36^b^
*L. rhamnosus* GG	41.30 ± 4.15	18.42 ± 3.10
*L. brevis* BK3	50.24 ± 3.12	14.37 ± 7.92

^a^Elastase inhibitory of oleanolic acid.

^b^Collagenase inhibitory of 1,10-phenanthroline.
